# RAId_DbS: mass-spectrometry based peptide identification web server with knowledge integration

**DOI:** 10.1186/1471-2164-9-505

**Published:** 2008-10-27

**Authors:** Gelio Alves, Aleksey Y Ogurtsov, Yi-Kuo Yu

**Affiliations:** 1 National Center for Biotechnology Information, National Library of Medicine, NIH, Bethesda, MD 20894, USA

## Abstract

**Background:**

Existing scientific literature is a rich source of biological information such as disease markers. Integration of this information with data analysis may help researchers to identify possible controversies and to form useful hypotheses for further validations. In the context of proteomics studies, individualized proteomics era may be approached through consideration of amino acid substitutions/modifications as well as information from disease studies. Integration of such information with peptide searches facilitates speedy, dynamic information retrieval that may significantly benefit clinical laboratory studies.

**Description:**

We have integrated from various sources annotated single amino acid polymorphisms, post-translational modifications, and their documented disease associations (if they exist) into one enhanced database per organism. We have also augmented our peptide identification software RAId_DbS to take into account this information while analyzing a tandem mass spectrum. In principle, one may choose to respect or ignore the *correlation *of amino acid polymorphisms/modifications within each protein. The former leads to targeted searches and avoids scoring of unnecessary polymorphism/modification combinations; the latter explores possible polymorphisms in a controlled fashion. To facilitate new discoveries, RAId_DbS also allows users to conduct searches permitting *novel *polymorphisms as well as to search a knowledge database created by the users.

**Conclusion:**

We have finished constructing enhanced databases for 17 organisms. The web link to RAId_DbS and the enhanced databases is . The relevant databases and binaries of RAId_DbS for Linux, Windows, and Mac OS X are available for download from the same web page.

## Introduction

Scientific literature, documenting different studies and conclusions, is among the most important sources of knowledge and biological information. It has been noted [1,2] that it is in the scientific community's best interest to be able to have such information consolidated and organized in an easy-to-use format so that researchers can integrate and/or interrogate the existing knowledge during biological data analysis. Such a knowledge integration may help researchers in identifying conflicting results [3], forming new hypotheses, and performing experimental validations. In the scope of proteomics studies to which we now turn, information related to single amino acid polymorphisms (SAPs) and post-translational modifications (PTMs) is among the most important.

Like single nucleotide polymorphisms (SNPs) that occur roughly every 300 base pairs [4], SAPs also differentiate individuals from one another. It is well known that SNPs may result in SAPs that are not yet annotated and thus not present in the standard protein databases. To enable identification of peptides containing this type of SAPs, Edwards [5] had come up with a compression scheme to reduce the size of the expressed sequence tag (EST) database to allow searches within the compactified database. In addition to resulting from nonsynonymous SNPs, however, SAPs may also occur due to post-transcriptional regulations such as mRNA editing [6]. SAPs together with PTMs are often used as disease markers [7-10]. Integration of this annotated, disease-related knowledge with data analysis facilitates speedy, dynamic information retrieval that may significantly benefit clinical laboratory studies.

**Figure 1:**
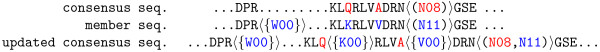
**Information-preserved protein clustering example**. Once a consensus sequence is selected, members of a cluster are merged into the consensus one-by-one. This figure illustrates how the information of a member sequence is merged into the consensus sequence. Amino acid followed by two zeros indicates an annotated SAP. Every annotated PTM has a two-digit positive integer that is used to distinguish different modifications. The difference in the primary sequences between a member and the consensus introduces *cluster-induced *SAPs. In this example, the residues Q and A (in red) in the consensus are different from the residues K and V (in blue) in the member sequence. As a consequence, K becomes a cluster-induced SAP associated with Q and V becomes a cluster-induced SAP associated with A. The annotated SAP, ⟨{W00}⟩, associated with residue R in the member sequence is merged into the consensus sequence, see the updated consensus sequence in the figure. Note that the annotated PTM, ⟨(N11)⟩, associated with N in the member sequence is merged with a different annotated PTM, ⟨(N08)⟩, at the same site of the consensus sequence. In this figure, all the merged information from the member sequence are shown in blue color to indicate that during the searches we can choose to respect the *correlated *information from each member sequence separately. To respect the correlated information means that when scoring the peptide segment LQ ⟨{K00}⟩ RLVA ⟨{V00}⟩ DR of the consensus sequence RAId_DbS only considers the combinations L(red Q)RLV(red A)DR and L(blue K)RLV(blue V)DR, but not L(red Q)RLV(blue V)DR and L(blue K)RLV(red A)DR. Having the choice to distinguish the SAPs/PTMs originated from individual member sequences, RAId_DbS can target on documented SAP/PTM combinations associated with certain disease (if it exists) and can avoid scoring unnecessary SAP/PTM combinations when there are several variable sites occurring within a peptide. However, currently we find almost no incidence of multiple variable sites within a short peptide in all our databases constructed. Therefore, the feature of respecting correlated information is only implemented in our in-house version, not yet in the web version. Furthermore, not forcing the integrity of correlated information also allows for novel SAP discovery in a controlled fashion, meaning that one is looking for SAPs with *local *precedence. Finally, let us emphasize that although the SAPs, PTMs are merged each annotation's origin and disease associations are kept in the processed definition file, allowing for faithful information retrieval at the final reporting stage of the RAId_DbS program.

To incorporate existing knowledge and information within peptide searches, we start by constructing a human protein database where information about annotated SNPs, SAPs, PTMs, and their disease associations (if any) are integrated. Consequently, the database part of our work may be considered an advancement of references [11] and [12]. The former extended the human protein database to include SAPs but without PTMs and without integration of disease information, while the latter allows for protein-specific annotated PTMs but without SAPs and without integration of disease information. We have also modified our peptide identification software RAId_DbS [13] to take into account the integrated information of annotated SAPs/PTMs and diseases while performing peptide searches. It is perceivable that the disease marker within a protein might be manifested as specific combinations of SAPs/PTMs, which we term information correlation. As explained in the caption of Figure 1, our database construction can easily accommodate correlations of this type. To further facilitate new discoveries, RAId_DbS allows users to conduct searches permitting *novel *SAPs.

However, it is worth pointing out that allowing annotated SAPs/PTMs (or novel SAPs) during the search, one is dealing with a larger search space than before and thus should anticipate an increase (decrease) in false positives (retrieval efficiency). Therefore, we recommend the users to turn on annotated SAPs/PTMs and novel SAPs only if the regular searches returns no significant hit. Specifically, we recommend the users to perform regular searches first. For spectra that do not receive any significant hit from regular searches, one may turn on annotated SAPs/PTMs and then search again. Finally, for spectra that receive no significant hit from both regular searches and searches with annotated SAPs/PTMs, one may turn on the novel SAPs together with annotated SAPs/PTMs and then search again.

We have built a web-based application taking query spectrum online as well as prepared standalone downloadable executables that can be installed locally on users' own machines. An important feature of the standalone version is the flexibility for users to add SAP and/or PTM information to various proteins they are interested in and even to create a user-specific database that contains new protein sequences. In the next section, we describe our database construction to illustrate how we accommodate the SAPs, PTMs, and their disease associations. We then provide a brief introduction to our software RAId_DbS and elaborate on its augmentation. In the result section, we use a few examples to show the structure of our database. The optimal use of our enhanced database in information retrieval is sketched in the discussion section.

## Database Construction

In the following discussion, we use human database construction as an example to illustrate how we enhance the protein databases. Similar procedures are employed to construct enhanced databases of other organisms, see Table 1 for a summary. We extracted 34,197 human protein sequences with a total of 16,814,674 amino acids from the flat file (last updated 09/05/2006) . Included in this file are proteins and their associated annotations generated respectively through the Reference Sequence and the Genome Annotation projects of the National Center for Biotechnology Information (NCBI). Each protein sequence is accompanied by a list of annotated SAPs and PTMs. Out of the 34,197 proteins, we found 29,979 unique proteins with a total of 15,324,913 amino acids. To avoid having multiple copies of identical or almost identical proteins in the database without losing information, we first perform an *information-preserved *clustering on the 34,197 sequences.

**Table 1:** Summary of Enhanced Organismal Databases Searchable by RAId_DbS

Organism	DB_name	Protein	NP	NM	SP	SAPs	PTMs	DB_size (byte)
*Homo sapiens*	hsa	29284	35059	35031	15030	116073	84406	16,265,018
*Anopheles gambiae*	angam	12388	12719	12706	112	350	50	6,042,277
*Arabidopsis thaliana*	artha	29651	31740	31711	5527	5207	11977	12,318,213
*Bos taurus*	botau	23796	26504	26491	3979	3295	15810	11,188,490
*Caenorhabditis elegans*	caele	22563	23097	23097	2890	1045	7756	10,050,609
*Canis familiaris*	cafam	31705	33834	33821	528	2766	4196	18,458,474
*Danio rerio*	darer	31192	36150	36137	1552	7358	3841	14,477,794
*Drosophila melanogaster*	drmel	17232	20207	20207	2568	5611	9290	9,796,785
*Equus caballus*	eqcab	17300	17637	17624	171	485	1045	9,404,150
*Gallus gallus*	gagal	18154	18724	18681	1455	1109	6522	8,728,501
*Macaca mulatta*	mamul	32547	38141	38128	207	1370	1262	14,498,187
*Mus musculus*	mumus	28506	35503	35451	12170	27614	61684	14,363,491
*Oryza sativa*	orsat	26636	26784	26777	1205	1291	2182	10,679,924
*Pan troglodytes*	patro	41464	52130	52117	482	3721	3734	20,217,986
*Plasmodium falciparum*	plfal	5240	5267	5267	88	56	184	3,995,386
*Rattus norvegicus*	ranor	28914	39425	39389	5569	9297	33240	15,879,569
*Saccharomyces cerevisiae*	sacer	5699	5880	0	5807	5507	13220	2,927,330

The header abbreviations in this table are explained as follows. The second column, headed by DB_name, documents the abbreviated database name for searches using standalone version of RAId_DbS. The column headed by "Protein" indicates the final number of protein clusters in the processed organismal databases. The columns headed by NP, NM, and SP summarize the breakdown of the total number of accession numbers included respectively from protein products, transcript products, and SwissProt protein entries. The columns headed by SAPs and PTMs indicate respectively the total number of annotated SAPs and PTMs included. The last column shows the database size in bytes.

### Information-Preserved Clustering (redundancy removal)

This process starts with an all-against-all BLAST [14] among the 34,197 sequences. Two sequences with identical lengths and aligned gaplessly with less than 2% mismatches are clustered, and each sequence is called a *qualified *hit of the other. Any other sequence that satisfies this condition with a member of an existing cluster is assigned to that existing cluster. All the annotations in the same cluster are then merged. We find it possible for every given cluster to choose a consensus sequence that will make all other members its polymorphous forms. Hence, we only retain one protein sequence for each of the 29,272 clusters finally obtained. The total number of amino acids associated with these 29,272 consensus proteins is 15,001,326. Although we only retain one sequence (the consensus sequence) per cluster, the information of other member sequences is still kept. For example, when a member sequence and the consensus sequence disagree at two sites, the presence of the member sequence is documented by introducing two *cluster-induced *SAPs at the two sites of the consensus sequence. The originally annotated SAPs and PTMs of the member sequence are also merged into those of the consensus sequence. Figure 1 and its caption illustrate how this process is done iteratively. In our processed definition file (see Table 2 for an example), each SAP or PTM is documented with its origin. SAPs arising from clustering are easily distinguished from annotated SAPs. For member sequences that are identical to the consensus sequence, the accession numbers of those member sequences are also recorded with their SAPs/PTMs annotations merged into the consensus sequence. When a user selects not to have annotated SAPs, RAId_DbS still allows for cluster-induced SAPs resulting in an effective search of the original databases but with minimum redundancy. The strategy employed by RAId_DbS to search for SAPs and PTMs will be briefly described in the "RAId_DbS and its Augmentation" section below.

**Table 2:** Example entries of the processed definition file

>*NP*_ 775259,*NM*_173167, *Q*8*IWX*7					
60	*I*00	*SAP*	*gTC *→ *A*	|*NM*_173167|	*dbSNP *: 16970659
60	*I*00	*SAP*	*v *→ *I*	|*Q*8*IWX*7|	*V *→ *I *(*dbSNP *: 16970659)*: FTId *= *V AR*_ 027506
199	*V *00	*SAP*	*GcA *→ *T*	|*NM*_173167|	*dbSNP *: 35749208
377	*R*00	*SAP*	*AaG *→ *G*	|*NM*_173167|	*dbSNP *: 41389545
496	*H*00	*SAP*	*d *→ *H*	|*Q*8*IWX*7|	*D *→ *H *(*breast cancersomatic mutation*). *FTId *= *VAR*_035870
778	*Q*00	*SAP*	*CgG *→ *A*	|*NM*_173167|	*dbSNP *: 34242925
852	*N*00	*SAP*	*AtC *→ *A*	|*NM_*173167|	*dbSNP *: 11654824
852	*N*00	*SAP*	*i *→ *N*	|*Q*8*IWX*7|	*I *→ *N *(*dbSNP *: 11654824). *FTId *= *V AR*_027507
>*NP*_076410,*NM*_023921,*Q*9*NY W*0					
92	*N*08,*N*09, *N*10, *N*11,*N*12			*PTM*	*Nlinked*(*GlcNAc*...) |*Q*9*NY W*0| *N *– *linked*(*GlcNAc*...)(*Potential*)
156	*M*00	*SAP*	*AcG *→ *T*	|*NM*_023921|	*dbSNP *: 597468
156	*M*00	*SAP*	*m *→ *T*	|*Q*9*NY W*0|	*M *→ *T *(*dbSNP *: 597468) *FTId *= *V AR*_030009
156	*M*00	*SAP*	*t *→ *M*	|*NP*_076410|	*Alignment with Q*9*NY W*0
158	*N*08,*N*09,*N*10, *N*11,*N*12			*PTM*	*Nlinked*(*GlcNAc*...) |*Q*9*NY W*0| *N *– *linked*(*GlcNAc*...)(*Potential*)

Two sequence clusters are shown in this table to demonstrate the structure of our processed information file. The text line after the ">" symbol contains accession numbers associated with the members of the cluster. The other rows each contains six entries separated by tabs. The first column indicates the residue position. The second column indicates the modified residue(s) that can occur at the position specified in the first column. The third column, labeled by either SAP or PTM, indicates the modification type. The fifth column contains the accession number of the source of modification, this may be a protein sequence or a mRNA. The fourth column explains the origin of the modification; a lower case letter indicates residue content in the source sequence, the upper case letter indicates the modified residue in the variant sequence. The notation, *v *→ *I*, indicates the source sequence with amino acid *V *can change into *I*, ie, a SAP. The notation, *gTC *→ *A*, is a short hand for codon change from *gtc *to *atc*, ie, a SNP that changes the coded amino acid from *V *to *I *as well. The sixth column contains additional information for the fourth column. It may include disease information or database entry index. As an example, in the first row of the first cluster, we have *dbSNP *: 16970659 indicating this SNP comes from the NCBI's dbSNP with entry index 16970659. In the fifth row, the sixth column contains disease origin. The additional Feature Identifier (FTId), VAR_xxxxxx, is the variant sequence documented by SwissProt. In the second cluster, fourth row, we see in the sixth column "Alignment with Q9NYW0", indicating that this SAP comes from the mismatch in the alignment between protein sequences in the clustering procedure. In the first and the last row of the second cluster, the second column contains N08, N09,..., N12, all of which are possible post-translational modifications associated with Glycosylations [22] as indicated in the sixth column.

### Ref_Seq Accession Number Retrieval

The consensus protein in a given cluster is then used as a query to BLAST against the NCBI's nr database to retrieve its RefSeq accession number and its corresponding Swiss-Prot  accession number, if it exists, from the best *qualified *hit. It is possible for a cluster to have more than one accession number. This happens when there is a tie in the qualified best hits and when a protein sequence in nr actually is documented with more than one accession number.

### Criteria for Inclusion of SAPs and PTMs

To minimize inclusion of less confident annotations, we only keep the SAPs and PTMs that are consistently documented in more than one source. For example, for proteins with Swiss-Prot accession numbers, we only keep the SAPs and PTMs that are present both in Swiss-Prot [15] and GenBank [16]. For proteins without Swiss-Prot accession numbers, the retentions of SAPs and PTMs are described below. The PTM annotations are kept only if they are present in the gzipped document HPRD_FLAT_FILES_090107.tar.gz of the Human Protein Reference Database . The SAP annotations are kept only if they are in agreement with the master table, SNP_mRNA_pos.bcp.gz (last updated 01/10/2007), of dbSNP: . Even though in many cases the primary information sources might be identical, the curation protocols may differ resulting in inconsistent annotations that are removed by our filtering strategy.

## RAId_DbS and its Augmentation

**Figure 2:**
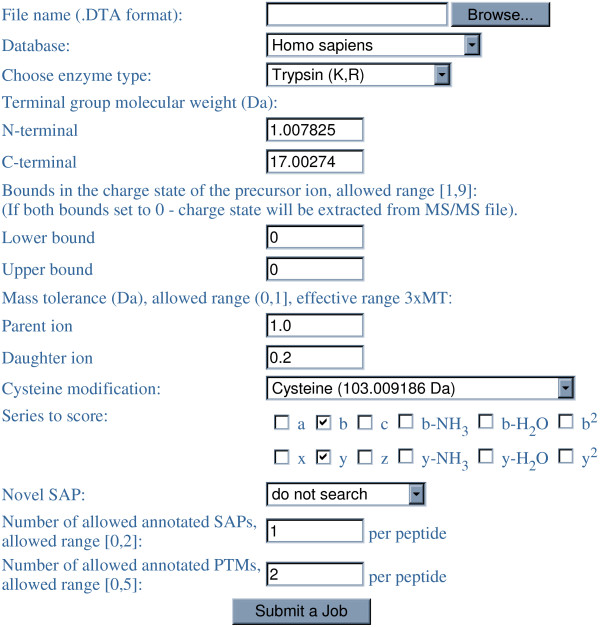
**RAId_DbS web interface**. The link to this webpage is . Enhanced databases for different organisms can be selected in the dropdown list.

**Figure 3:**
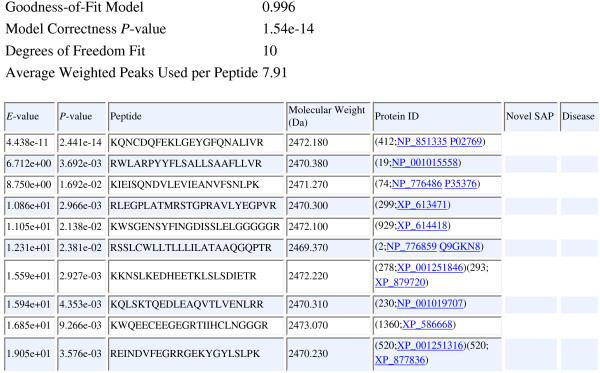
**The Format of Search Results Reported by RAId_DbS**. The report format of RAId_DbS contains a header portion that shows important relevant information pertinent to the score statistics. The Goodness-of-fit of the score model is reported. Basically, it represents how well the score model, be theoretically derived or assumed, agrees with the cumulated score histogram. Also reported is a quantity called the model *P*-value, which documents the likelihood for the correlation strength between the model score distribution and the score histogram to come out of random matching. In the report table, the first column shows the *E*-values and the second column shows the *P*-values. The protein IDs, shown in fifth column, also serves as the links to the proteins containing the reported peptide. The sixth column contains information of novel SAPs, if the reported peptide contains a novel SAP. The seventh column shows disease information, if the reported peptide contains disease related SAPs or PTMs.

The web interface of RAId_DbS is shown in Figure 2. An example of the reported peptide list resulting from a search is shown in Figure 3. Note that the Goodness-of-fit of the score model is reported. Basically, it represents how well the score model, be theoretically derived or assumed, agrees with the cumulated score histogram. Also reported is a quantity called the model *P*-value, which documents the likelihood for the correlation strength between the model score distribution and the score histogram to come out of random matching. Readers are referred to reference [13] for details.

### RAId_DbS Statistics

Here we briefly explain the underpinning of RAId_DbS statistics and RAId_DbS's strategy to deal with searches in different effective database sizes. The latter strategy can be generalized to handle effective database size expansion due to inclusion of SAPs and PTMs. Taking into account the finite sample effect and the skewness of peak intensity distribution, the form of asymptotic score statistics (*P*-values) of RAId_DbS [13] is *derived theoretically*. Since the skewness varies *per spectrum*, the theoretically determined parameters for our derived distribution are *spectrum-specific*. For most spectra considered, our theoretical distributions (used to compute *P*-values) agree well with the score histograms accumulated. The final *E*-value for each peptide hit in a search, however, is obtained by multiplying the peptide's *P*-value by the number of peptides of its category. As a specific example, when trypsin is used as the digesting enzyme, RAId_DbS allows for incorrect N-terminal cleavages. RAId_DbS has internal counters, *C*_*c *_and *C*_*inc*_, totaling respectively the number of scored peptides with correct and incorrect N-terminal cleavage. In general, *C*_*inc *_≫ *C*_*c*_. When calculating the *E*-value of a peptide with correct N-terminal cleavage, RAId_DbS multiplies the peptide's *P*-value by *C*_*c*_. However, the *E*-value of a peptide with incorrect N-terminal cleavage will be obtained by multiplying the peptide's *P*-value by *C*_*c *_+ *C*_*inc *_[13]. In line with the Bonferroni correction that is rooted in the Bonferroni inequality [17], our approach avoids overstating the significance of a hit from a larger effective database (the pool of peptides regardless of whether the N-terminal cleavage is correct) versus a hit from a smaller effective database (the pool of peptides with correct N-terminal cleavage only).

### RAId_DbS Augmentation

The same statistical approach is used in the augmented RAId_DbS. Different counters are set up to record the numbers of scored peptides in different categories. As a specific example, when novel SAPs are allowed, RAId_DbS creates a new counter, *C*_*novel_sap*_, to total the number of scored peptides with a novel SAP. This is in general a much larger number than other counters. When we calculate the *E*-value associated with a peptide hit that contains a novel SAP, we multiply the peptide's *P*-value by the sum of existing counters with *C*_*novel_sap *_included. However, in the same search, for a peptide without novel SAP, its *E*-value is obtained by multiplying the peptide's *P*-value by the sum of existing counters *excluding C*_*novel_sap*_. The same approach is applied to PTMs and other annotations.

**Figure 4:**
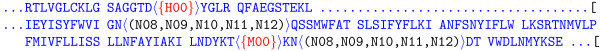
**Structure of Enhanced Database**. Consensus protein sequences NP_775259 (first line, residues 480 – 510 shown) and NP_076410 (second and third lines, residues 81 – 170 shown) are used as examples to demonstrate the structure of our sequence file, part of the enhanced database. A "[" character is always inserted after the last amino acid of each protein to serve as a separator. Annotated SAPs and PTMs associated with an amino acid are included in a pair of angular brackets following that amino acid. SAPs are further enclosed by a pair of curly brackets while PTMs are further enclosed by a pair of round brackets. Amino acid followed by two zeros indicates an annotated SAP. Every annotated PTM has a two-digit positive integer that is used to distinguish different modifications.

Below we briefly sketch how RAId_DbS deals with the presence of annotated SAPs, PTMs as well as novel SAPs. In our database format, annotated SAPs and PTMs are inserted right after the site of variation, see Figures 1 and 4. From this point on, we will call sites containing annotated SAPs/PTMs variable sites and sites without annotated SAPs/PTMs unvaried sites. When searching the database for peptides with parent ion mass 1500 Da, for example, RAId_DbS sums the masses of amino acids within each possible peptide to see if the total mass is within 3 Da (the default parent ion mass error range of RAId_DbS) of 1500 Da. At this stage, a variable site has, instead of a fixed mass, several possible masses depending on the number of annotated SAPs/PTMs at that site. A peptide that covers some variable sites therefore has several masses, each corresponding to a specific arrangement of SAPs/PTMs. If some of these masses happen to be within 3 Da of 1500 Da, RAId_DbS will score this peptide with corresponding annotated SAPs/PTMs that give rise to the proper masses. If none of these masses are within the allowed molecular mass range, that peptide will not be scored. It is worth pointing out that the default mass error tolerance (3Da) may be changed by the user on the web page while submitting a query spectrum to search.

Note that our approach is computationally efficient in terms of mass selection. For example, if a peptide contains a variable site, one first sum the amino acid masses of unvaried sites to obtain *m*_*unv*_. One then checks whether the masses associated with the variable site adding to *m*_*unv *_will fall in the desirable mass range. This approach is particularly powerful when there is more than one variable site in the peptide considered. As demonstrated in Figure 1 and its caption, the combinatorics associated with two variable sites result in only a longer list of possible masses to be added to *m*_*unv*_. This should be contrasted with methods that incorporate SAPs via appending polymorphous peptides to the end of the primary sequence. In the latter approach, the program needs to do the mass sum multiple times, repeating the mass sum of unvaried sites, and thus may slow down the searches.

Despite RAId_DbS's strategic advantage, introduction of SAPs/PTMs does increase the complexity of the algorithm. Therefore, during the searches RAId_DbS only considers for each candidate peptide to have up to two annotated SAPs and up to five annotated PTMs. To facilitate discovery, RAId_DbS also permits novel SAPs, but limited to one novel SAP per *not-yet-annotated *peptide, meaning peptides that do not contain any annotated SAPs/PTMs. This is because the introduction of novel SAP largely expands the search space, and if one allows novel SAPs within peptides already documented with SAPs/PTMs, the search space expansion will be even larger and may render the search intractable. Currently, the novel SAP search is expedited via a pre-computed list of amino acid mass difference. As an example, assume that one is searching for a peptide with parent ion mass 1500 Da, and a not-yet-annotated candidate peptide has mass 1477 Da, 23 Da smaller than the target mass. It happens that 23 Da is also the mass difference between Tryptophan and Tyrosine, and if the candidate peptide contains a Tyrosine, RAId_DbS will replace that Tyrosine with a Tryptophan and score the new peptide. If the candidate peptide contains two Tyrosines, RAId_DbS will replace one Tyrosine at a time with a Tryptophan and score both of the new peptides. It is evident that the complexity grows fast if one were to allow for two novel SAPs or more per peptide.

## Results and Analysis

In this section, we first report the status of our ongoing construction of and real examples of enhanced organismal databases. Comparison to related approaches will also be provided, followed by a few example studies.

### Database Construction Status

As summarized in Table 1, we have finished constructing databases for 17 organisms. Note that disease information is included only in the human database. Within the enhanced human database, we have 123,464 SAPs and 81,984 PTMs. Of those SAPs and PTMs, 15,787 have disease associations. In each enhanced organismal database, the consensus sequences (after information-preserved sequence clustering) are fused into a single string separated by the "[" character. This long string is stored in a file with a suffix ".seq" or simply called the sequence file. The sequence identifiers and other annotations are relegated into a file with a suffix ".def" or called a *processed *definition file. The processed definition file is only used in the final reporting stage of the search. A typical protein sequence in an enhanced sequence file carries with it annotated SAPs and PTMs in a simple format. In Figure 4 we show two consensus sequences containing SAPs/PTMs. The entries, associated with these two consensus sequences, in the processed definition file are shown in Table 2.

**Figure 5:**
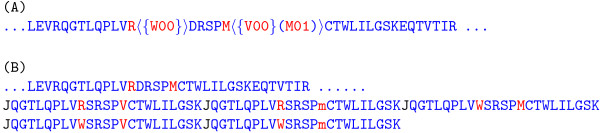
**Illustration of Minimum Redundancy of our Database**. In this example, the sequence has two nearby variable sites with residues R and M colored in red. Residue R may be replaced by a residue W due to a possible SAP; while residue M may be replaced by a residue V or an acetylated methionine (M01, in our notation) due to respectively a possible SAP or PTM. This information is encoded in our sequence file as shown in part (A). To encode the same information, method proposed in reference [11] would have up to five additional highly similar peptides separated by a letter "J" appended to the end of the primary sequence, see part (B). Here a lower case m is used to denote the acetylated methionine. Another key difference in the two methods shown above is on the limit of allowed number of enzymatic miscleavages. In our method, there is no limit on the number of allowed miscleavages, while in other approaches, the number of miscleavages is usually set to below a certain threshold. As an example, in our method, the variant peptides SPVCTWLILGSKEQTVTIR and SPmCTWLILGSKEQTVTIR are already included in (A). But in the approach of reference [11], in order to allow consideration of this variant peptide, one either needs to additionally append this peptide or to have much longer flanking peptides than shown in (B).

The format of our sequence file minimizes redundancy in searches. For example, if a single site contains two SAPs, construction method proposed by Schandorff *et. al*. [11] will demand two almost identical partial sequences, each may be several tens of amino acids in length, be appended after the primary sequence, while in our case it only takes up a few additional bytes. The compactness of our database becomes obvious when incorporating the information of two nearby sites, each containing several annotated SAPs and PTMs, into the database. In our construction, we only need a few additional bytes. But in other approaches, it may introduce an appreciable expansion due to including/excluding and pairing of different variations at both sites along with the flanking peptides, see Figure 5 for an illustration. Another key difference between our method and other database methods is that we do not need to limit the number of enzymatic miscleavages.

**Figure 6:**
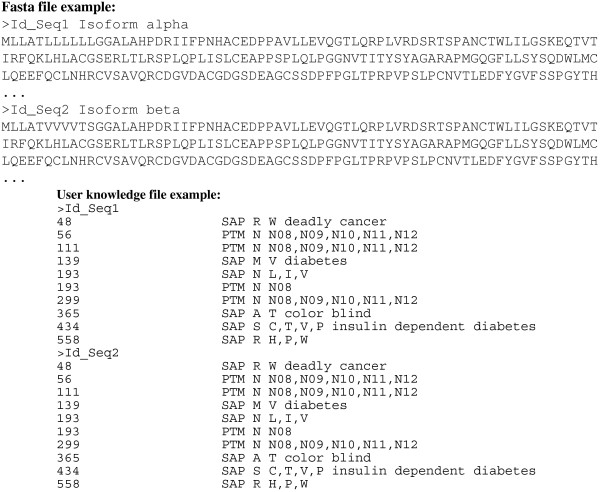
**An Example of user-specific database construction**. To construct a user-specific database, the user needs to provide a FASTA file containing sequences of interest and a flat information file documenting the SAPs/PTMs and disease information that the user wishes to consider. In this example, Id_Seq1 and Id_Seq2 represent sequence identifiers. In the information file, the format is as follows. First column indicates residue position; second column specifies whether the modification is a SAP or PTM; third column records the original residue in the sequence at position specified in the first column; fourth column consists of either a list of possible SAPs (L, I, V) or a list of possible PTMs (N08, N09, N10, N11, N12); fifth column documents disease names, if any, associated with the modifications at the specified positions. The user may then run our script, UserDb.pl, to generate the appropriate ".seq" and ".def" files suitable for searching using RAId_DbS. More detail can be found in the help page  of RAId_DbS.

When needed and when using the standalone version of RAId_DbS, users may create their own databases with user-specific knowledge input. The user will provide both a FASTA file containing sequences to be included and a separate information file documenting the modifications and annotation associated with variable sites of each sequence. The format is illustrated in Figure 6. Through our Perl script UserDB.pl, the flat information file -containing the protein accession numbers, detailed SAP/PTM information, and disease associations- is processed together with the FASTA file provided by the user to generate the user-specific ".seq" file and ".def" file which are suitable for searches using RAId_DbS. If one wishes to add additional SAPs or PTMs, one simply updates both the FASTA sequence file as well as the flat information file and rerun the Perl script. When reporting a hit with annotated SAPs or PTMs, RAId_DbS automatically reports the corresponding detailed information and disease association if it exists.

### Examples

Using a tandem mass (MS^2^) spectrum taken from the profile dataset described in reference [18], we illustrate in Table 3 two search results in the human protein database with the annotated SAPs and PTMs turned off (a) and on (b) respectively. In case (a), the best hit is a false positive with *E*-value about 0*:*11 implying that one probably ends up declaring no significant peptide hit for this spectrum. In case (b), however, the best hit is a true positive (a peptide from human *transferin *with an annotated SAP) with *E*-value about 4.0 × 10^-7^. This example shows that if properly used, allowing SAPs/PTMs may increase the peptide identification rate. That is, it may be fruitful to turn on the SAPs/PTMs when a regular search returns no significant hit. Turning on SAPs/PTMs without first searching with SAPs/PTMs turned off, however, may cause a loss of sensitivity due to the increase of search space.

**Table 3:** Example search results of augmented RAId_DbS

(a) *E*-value	*P*-value	Peptide	Mol. Wt.	Protein ID	Novel SAP	Disease
1.184e-01	1.744e-05	RTKLKDC...KIAR	2897.500	(NP_114412;...;Q9H2L5)	disabled	
⋮	⋮	⋮	⋮	⋮	⋮	⋮
4.084e+00	9.345e-03	KQQELAA...VSSR	2898.520	(NP_072096;...;O75420)	disabled	

(b) *E*-value	*P*-value	Peptide	Mol. Wt.	Protein ID	Novel SAP	Disease

3.977e-07	1.834e-10	KsVEEYANCHLAR	1448.650	(NP_001054;...;P02787)	disabled	
4.779e-01	2.205e-04	KsVqEYANCHLAR	1447.670	(NP_001054;...;P02787)	disabled	
⋮	⋮	⋮	⋮	⋮	⋮	⋮
7.524e-01	3.470e-04	RℓMNAsMVWAQAAR	1448.720	(NP_000337;...;P48436)	disabled	{(ℓ;2;108;Campomelic dysplasia (CMD1) [MIM:114290])(s;6;...)}

In this example, for the spectrum considered, RAId_DbS performs searches assuming the parent ion to have charge +1, +2, +3. The search results are then pooled together to form a single result ranked by *E*-values. This is why for the same spectrum RAId_DbS may report peptide hits with very different masses. In part (a) ((b)), searches were done with annotated SAPs and PTMs turned off (on). The lowercase letters in the peptide indicate SAPs. A novel SAP, if present and enabled in the searches, will be specified in the column headed by Novel SAP. Note that in the disease related annotation, there are four fields separated by three semicolons. The first field, a lower case amino acid letter, indicates the SAP; second field, an integer, indexes the SAP position in the peptide; the third field, an integer, indexes the SAP position in the protein; the fourth field shows the annotated disease association.

It is commonly believed that when searching a large database, sensitivity is severely lost. This is particularly true if the *E*-value for every hit is obtained by multiplying the peptide's *P*-value by the same number (*e.g*. the largest effective database size) regardless of the category that peptide belongs to. As we have explained earlier, RAId_DbS does not do that. It uses a method equivalent to Bonferroni correction. We use *E*-values to rank peptide hits and each peptide's *E*-value is obtained by multiplying its *P*-value by the corresponding size of the effective database that the peptide belongs to. Consequently, to be equally significant, peptide hits falling in a category that has a large effective database size need to have smaller *P*-values than those of peptide hits falling in a category that has a small effective database size.

Nevertheless, even with such a strategy one can never guarantee to bypass the sensitivity loss problem associated with searching a large database.

Although we have shown [19] as a preliminary study that no appreciable loss of sensitivity is found using the 54 training spectra of PEAKS [20], the number of spectra there is too small to ensure our observation to be statistically robust. We therefore set out to use spectra from a larger collection of human proteins [21], henceforth referred to as the Aurum dataset, to test the severity of sensitivity loss when expanding the search space via turning on SAPs/PTMs and novel SAPs. The Aurum data [21] is a set of MS/MS spectra generated in an ABI 4700 MALDI TOF/TOF instrument. The sample is a mixture of 246 human proteins that were individually purified and tryptically digested. This data was developed to be a standard reference for the purpose of testing or training new algorithms.

**Figure 7:**
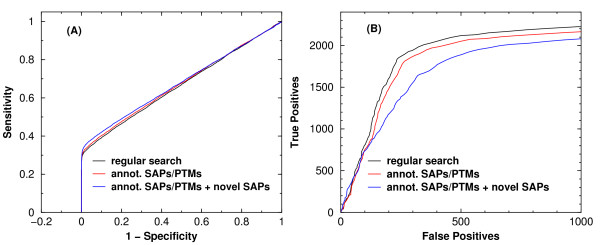
**ROC curves obtained from different search modes**. ROC curves for three different search modes employed when running RAId_DbS using the Aurum dataset composed of 9977 spectra. In panel (A) curves are shown in sensitivity versus (1-specificity), while in panel (B) the cumulative number of true positives versus the cumulative number of false positives are shown. In panel (B), the increase in the number of false positives coming from annotated SAPs/PTMs (red curve) and with novel SAPs (blue curve) is anticipated due to a larger search space compared to searches done within the standard database only. The larger total number of false positives found in the latter methods, however, will push the ROC curves leftwards upon normalizing to 1-specificity.

In Figure 7, we show the Receiver Operating Characteristic (ROC) curves when analyzing the Aurum dataset [21] which contains 9977 spectra from a selection of human proteins. In each of the two panels of Figure 7, there are three ROC curves corresponding to *regular *searches without SAPs/PTMs, searches allowing annotated SAPs/PTMs, and searches allowing both annotated SAPs/PTMs as well as novel SAPs. Although there seems to be no performance degradation judging from the sensitivity versus 1-specificity plot (panel (A)), we do see mild degradation in terms of the cumulative number of true positives found using a fixed number of false positives as the threshold (panel (B)). Compared to regular searches, turning on SAPs/PTMs and novel SAPs results in a larger number of false hits which pulls the ROC curve towards the left end upon normalization. This may partially explain why turning on SAPs/PTMs and novel SAPs does not introduce an appreciable loss in sensitivity.

## Discussion

From analyzing the Aurum dataset using different search spaces, we confirmed that the number of true positives found at a false positive threshold may decrease if the search is done in a larger search space, i.e., with novel SAPs and/or annotated SAPs/PTMs unconditionally enabled. This indicates that it is not productive to search with the annotated SAPs/PTMs and novel SAPs enabled all the time. We recommend the user to turn on these features conditionally. For example, if a spectrum does not receive any significant hit from a regular search, one may then allow the annotated SAPs/PTMs. If the search still returns no significant hit, one may then turn on novel SAPs in the search. It is in this context that one may increase the number of peptides identified.

It has not escaped our attention that the ROC curves shown in panel (B) of Figure 7 do not rise steeply as one typically sees. This, however, may be caused by the presence of contaminants during protein purification that introduced peptides not belonging to the target proteins. Since our main purpose is to study the *relative *sensitivity degradation upon enlarging the search space, we do not delve into the investigations of peptide hits with low *E*-values but not subsequences of target proteins.

The processed definition files associated with our enhanced databases contain consolidated information in a tab delimited format, allowing easy information extraction by others who are interested in utilizing this information in different contexts. While the information contained in our enhanced databases are helpful in terms of forming hypotheses and narrowing down the scope of investigation, it should be used with caution because scientific literature, consisting of coherent information, also contains conflicting information. Therefore the reported disease association should not be used as a diagnosis report but only be used as a reference for further investigation. In particular, from clinical application point of view, patients and clinical scientists may benefit from such information as it suggests possibilities of diseases that may otherwise be overlooked.

It is our plan to continue construction of enhanced databases for additional organisms. Although little disease information exists for most organisms other than human, we will include it in our databases when more information becomes available. For example, the NCBI's Online Mendelian Inheritance in Animals OMIA,  contains information of genes, inherited disorders and traits in animal species (other than human and mouse). We plan to integrate this information into our organismal databases in the near future. Under our database format, it is also possible to incorporate other information such as protein fusions, 3D protein structures, drug-binding/active sites, cross-linking sites, and isoforms. We are currently assessing which additional information to include next. It is also worth pointing out that our database compactification via clustering has an advantage in reducing search time.

Without collapsing identical and almost identical proteins, one is bound to score identical peptides multiple times. Our compactification strategy minimizes redundant searches of this sort. This reduction of redundancy will become important when exploring unrestricted PTM searches.

## Authors' contributions

All authors contributed to the design of the research and analysis of the results, GA and AO carried out the research, YKY wrote the paper. All authors read and approved the final manuscript.
